# PDP type brain tumor in association with multiple endocrine neoplasia type 1

**DOI:** 10.1016/j.heliyon.2024.e27418

**Published:** 2024-03-12

**Authors:** Halldór Bjarki Einarsson, Anja Lisbeth Frederiksen, Inge Soekilde Pedersen, Marianne Schmidt Ettrup, Martin Wirenfeldt, Henning Boldt, Nina Nguyen, Marianne Skovsager Andersen, Carsten Reidies Bjarkam, Frantz Rom Poulsen

**Affiliations:** a Department of Neurosurgery, Aalborg University Hospital, Denmark; b Molecular Diagnostics, Aalborg University Hospital and Clinical Cancer Research Center, Aalborg University Hospital, Denmark; c Department of Clinical Medicine, Aalborg University, Denmark; d Department of Pathology, Aalborg University Hospital, Denmark; e Department of Pathology, Hospital South West Jutland, Denmark; f Department of Regional Health Research, University of Southern, Denmark; g Department of Pathology, Odense University Hospital, Denmark; h Department of Neuroradiology, Odense University Hospital, Denmark; i Department of Endocrinology, Odense University Hospital, Denmark; j Department of Neurosurgery, Odense University Hospital, Denmark; k Department of Clinical Research and BRIDGE, Brain Research – Inter-Disciplinary Guided Excellence, University of Southern, Denmark

**Keywords:** Ependymoma, Multiple endocrine neoplasia type 1, Pleomorphic xanthoastrocytoma, Cyclin-dependent kinase inhibitor

## Abstract

Multiple endocrine neoplasia type 1 (MEN1) is a rare autosomal dominant syndrome caused by inactivating pathogenic variants in the tumor suppressor gene menin 1 on chromosome 11q13 (Falchetti et al., 2009). The syndrome is characterized by neoplasia in two or more endocrine glands and has a high degree of penetrance. Pathogenic germline multiple neoplasia type 1 variants primarily result in neoplasia affecting the parathyroid glands, the pancreatic islet cells, and the anterior pituitary in combination. Primary hyperparathyroidism is the most common pathological manifestation of the syndrome, followed by pancreatic neuroendocrine tumors. Important genetic confirmation has been provided showing that ependymoma should be considered as a neoplasm that can occur in patients with MEN1 (Kato et al., 1996; Cuevas-Ocampo et al., 2017). The biphasic histopathological tumor entity shown in the present case we name Pleomorphic Xanthoastocytoma grade 3 differential pathology (PDP) in association with Multiple Endocrine Neoplasia type 1. This MEN1 associated tumor subtype is an extension of the findings on MEN1 associated ependymoma, where we show that the clinical phenotype itself may potentially be triggered by a frameshift germline pathogenic variant for the MEN1 gene, in combination with cyclin-dependent kinase inhibitor 1B gene germline variant and cyclin dependent kinase inhibitor 2A somatic deletion downstream of menin.

## 1 Introduction

The case presented reveals potential new genetic aspects of Pleomorphic Xanthoastocytoma (PXA) grade 3 as part of multiple neoplasia type 1 (MEN1) syndrome [[1], [2], [3]]. MEN1 is a rare autosomal dominant syndrome caused by inactivating pathogenic variants in the tumor suppressor gene menin 1 gene (*MEN1*) on chromosome 11q13. The syndrome is characterized by neoplasia in two or more endocrine glands and has a high degree of penetrance [4]. Pathogenic germline *MEN1* variants primarily result in neoplasia affecting the parathyroid glands, the pancreatic islet cells, and the anterior pituitary in combination. Primary hyperparathyroidism is the most common pathological manifestation of the syndrome, followed by pancreatic neuroendocrine tumors [5,6]. In 2017, Cuevas-Ocampo et al. provided genetic confirmation that ependymoma should be considered as a neoplasm that can occur in patients with MEN1 syndrome [3]. In the present case, we extend their work by showing that the clinical phenotype itself may potentially be triggered by a frameshift germline pathogenic variant in *MEN1* in combination with cyclin-dependent kinase *(CDK)* inhibitor 1B gene (*CDKN1B*) germline variant and *CDKN2A/B* somatic deletion, downstream of menin. Pathogenic germline *CDKN1B* variants associate with the rare MEN4 syndrome that presents with similar phenotypes to MEN1 [7]. *CDKN1B* is transcriptionally upregulated by menin, and *CDKN1B* variants are reported to be associated with a higher risk of multiple tumors in MEN1 patients [[8], [9], [10]]. Therefore, genetic variants or altered gene expression downstream of *MEN1*, *e.g. CDKN1B* or in interacting signaling pathways, are hypothesized to modify the phenotypes. However, it is unknown whether patients with MEN1 and ependymomas or PXA have additional pathogenic variants or polymorphisms in genes that regulate downstream signaling pathways.

### 1.1 Case presentation

**Fig. 1:**
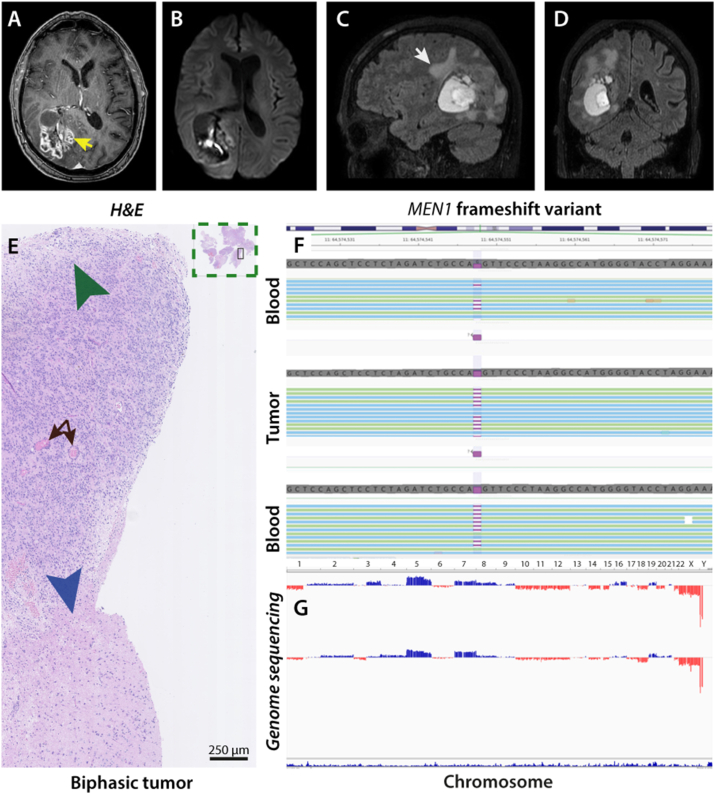
a Contrast-enhanced axial T1-weighted MRI showing heterogenous contrast-enhancing tumor abutting the right ventricular atrium and posterior horn (yellow arrow). **b** DWI-weighted imaging revealing infiltration of the tumor into the ventricular system. **c** Sagittal plane with FLAIR sequence showing edema (white arrow). **d** Coronal plan indicating mass effect with total occlusion of the right posterior horn of the ventricular system. **e** H&E-stained tumor biopsy demonstrating biphasic tumor. Above the biphasic border (blue arrow), key histopathological features of ependymoma WHO grade III can be detected, partly infiltrative high cellularity tumor (green arrow) and with perivascular pseudorosettes (black arrows). Beneath the border (blue arrow), pleomorphic xanthoastrocytoma-like features with spindle cells and lipid-laden xanthomatous astrocytes (magnification × 10). **f-g** Identification of a pathogenic germline variant, *MEN*1, NM_130799.2:c.847delC, p.(Leu283Trpfs*4) in the heterozygous state. The variant was present in most (77%) of the sequence reads from the tumor samples. Shallow whole-genome sequencing of the two tumor samples displaying multiple chromosomal aberrations including deletion (red bars) of chromosome 11 harboring *MEN1*.

The male proband was a 53-year-old Caucasian man who had presented with primary hyperparathyroidism (PHP) 21 years earlier in 1992 and had undergone resection of two parathyroid adenomas; postoperatively, the patient had hypocalcemia. Since then, recurrent deep venous thromboembolism (DVT) had necessitated lifelong warfarin therapy. The family history included MEN1 in the mother and the son, and the suspicion of a MEN1 diagnosis was raised in the proband due to nephrolithiasis and PHP. The MEN1 diagnosis was genetically verified with the identification of MEN1, c.847delC, p.(Leu283Trpfs*4), and family members have not been screened for the same CDKN1B germline variant. At the first consultation, the proband presented with left homonymous hemianopsia confirmed by computer perimetry. In addition, exophoria was detected with induced diplopia. Besides hemianopsia and intermittent diplopia, the patient complained of headache with maximal intensity in the right parietooccipital region, and left side hemisensory disturbances were present. Neural axis magnetic resonance imaging (MRI) was performed, and the scan revealed a contrast-enhancing multilobular tumor mass involving the right precuneus, primary and accessory sensory cortex, and striate area in close approximation to the ependyma of the right ventricular system (Fig. 1a–d).

The primary tentative diagnosis was oligodendroglioma as the tumor was thought to represent a grade III oligodendroglioma based on the MRI characteristics. Acute administration of 100 mg oral prednisolone was indicated due to detection of edema in the periphery of the tumor process on the FLAIR and T2W sequences. Despite a Glasgow Coma Scale score of 15, subacute surgery was planned because of the tumor size and the severity of tumor infiltration. Warfarin was paused and although the international normalized ratio (INR) blood test at the time was 5.3 (therapeutic range 2.0–3.0), there was no postponing of surgery; instead, prothrombin complex concentrate therapy with Octaplex 3000 IE was given intravenously at an infusion rate of 1 ml/min. The day after, with INR at 1.1, tumor resection was performed under guidance of the S8 stealth navigational system and fluorescein microsurgery. The same prednisolone dose was given postoperatively for two days and then gradually reduced, and warfarin was reinitiated on the third postoperative day, i.e. after the 48-h early postoperative MRI. Postoperative imaging identified no contrast-enhanced tumor remnant or hemorrhage.

At follow-up one month later and after the first proton therapy, the patient presented with the same neurological deficits as preoperatively, and with complete remission of headache. At this clinical consultation, a lumbar puncture for cerebrospinal fluid (CSF) cytology was postponed due to high INR (5.8). Despite this elevated INR, there were clinical signs of DVT in the left arm; these were confirmed by acute ultrasound scanning that revealed a DVT in the left brachial vein. The patient was treated successfully with 18000 IE Fragmin, and warfarin was stopped in favor of lifelong novel oral anticoagulant (NOAC) treatment. Specific research on potential genetics revealing the genesis of the coagulopathy and recurrent DVT was not carried out, and the DVT recurrence was considered due the tumor load or the cancerous condition [11,12]. Lumbar puncture was performed one week later, and no shedding of tumor cells or pathogens were detected in the CSF. Primary histopathology indicated that the resected tumor was an anaplastic ependymoma (ependymoma WHO grade III). However, because the tumor was histologically biphasic (Fig. 1e) and the methylation profile did not provide substantial information compatible with a known histological diagnosis, the tumor was screened for germline and somatic genetic variants using whole-exome sequencing (WES).

DNA was extracted from two source types, i.e. from lysed leucocytes in peripheral blood and from formalin-fixed paraffin-embedded (FFPE) tumor tissue, using two different FFPE blocks. Estimated neoplastic cell content was 80% (block #1) and 90% (block #2). Screening for germline and somatic genetic variants was conducted using WES, with analyses confined to *MEN1* and *CDKN1B*. Library preparation was performed using SureSelect XT HS and Clinical Research Exome v2 capture kit (Agilent) according to the manufacturer's protocol. Libraries were sequenced on a NovaSeq 6000 (Illumina) with 2x150 bp reads. An in-house pipeline based on Burrows-Wheeler Aligner (BWA) and the Genome Analysis Toolkit GATK4.0 were used for mapping and variant calling. Subsequent analyses were confined to *MEN1* and *CDKN1B.* Mean coverage was 190 X, and 100% of the coding regions were covered >30 X. To detect large copy number variations (CNVs) such as chromosomal deletions and duplications, shallow whole-genome sequencing was performed as previously described on DNA extracted from FFPE ependymoma tissue [13].

For the Illumina Infinium Methylation EPIC bead chip array analysis and CNS tumor panel Next-Generation Sequencing (NGS)*,* DNA was purified from 10 μm paraffin sections using GeneRead DNA FFPE Kit (Qiagen, Hilden, Germany) according to the manufacturer's instructions and as described previously [14,15]. EPIC bead chip array analysis collects data on the DNA methylation status of >850,000 CpG sites. DNA quantitation was performed on Qubit 2.0 with Qubit® dsDNA HS Assay (Thermo Fisher Scientific, Waltham, MA, USA). DNA quality was assessed by qPCR with SYBRGreen PCR Master Mix on QuantStudio 12K Flex Real-Time PCR System (Applied Biosystems, Foster City, CA, USA) based on the FFPE QC Kit (Illumina, San Diego, CA, USA). Bisulphite conversion was performed using Zymo EZ DNA Methylation kit (Zymo Research, Irvine, CA, USA). The bisulphite-converted DNA was restored using Infinium HD FFPE Restore Kit from Illumina and processed with ZR-96 DNA Clean & Concentrator-5 kit (Zymo Research, Irvine, CA, USA). The EPIC bead chip array was prepared according to the manufacturer's instructions, and data were collected with the iScan instrument (Illumina). The EPIC bead chip array and associated reagents and solutions were from Illumina, USA. Brain tumor classification and generation of copy number variation (CNV) profile was performed in silico using the MolecularNeuropathology.org server (v11b4 and v12.5) as described by Capper et al. [16]. Sequencing was performed using a panel of 20 genes frequently mutated in brain tumors [17], in combination with the Ion Torrent S5 System and associated reagents (Thermo Fisher Scientific, Waltham, MA, USA). Following primary annotation using standard Ion Torrent Suite workflows (Mapped Reads 1,763,784; Mean Depth: 2693; Uniformity: 95.24%), secondary annotation of VCF files was performed with Ion Reporter, while GenomeBrowse (Golden Helix, Bozeman, MT, USA) was used for manual assessment of BAM alignment files. The sequencing experiment included a control sample from a healthy donor.

The pathogenic germline variant, *MEN*1, NM_130799.2:c.847delC, p.(Leu283Trpfs*4) in the heterozygous state was identified (Fig. 1f and g). This frameshift variant located in exon 6 results either in a substantially truncated protein or no protein production at all. The variant is not found in population databases (gnomAd exomes and gnomAd genomes). As it is a null variant in a gene for which loss-of-function is a documented mechanism of disease, the variant is classified as pathogenic according to ACMG guidelines [18].

In *CDKN1B,* the germline variant NM_004064.4:c.-79T > C (rs34330) in the heterozygous state was detected in the promoter region. This is a variant frequently reported in population databases. The T allele has been associated with cancer susceptibility [10,19], and *in vitro* it confers lower transcription rate of *CDKN1B* [20].

**Fig. 2:**
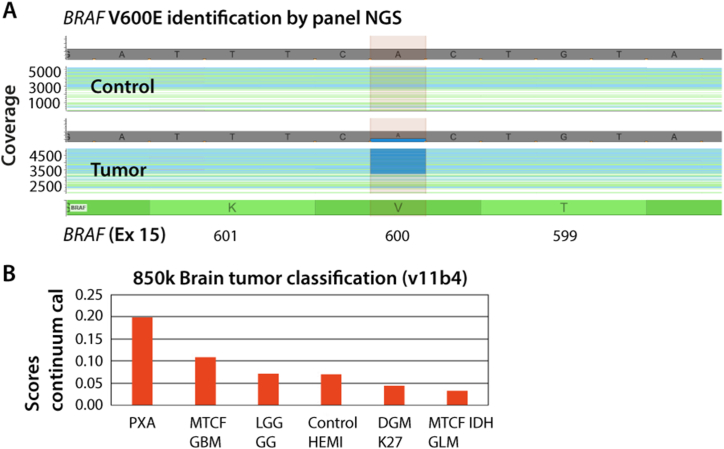
a Focused Next-Generation Sequencing (NGS) using a panel with 20 genes commonly mutated in gliomas revealed a somatic BRAF p.Val600Glu (V600E) variant with frequency of 33%. Coverage of sequencing raw data visualized by a genome browser that depicts the variant in blue. A healthy control, shown in the upper panel, was included for comparison. **b** DNA methylation profiling using 850k array data was unable to match the tumor to a known entity, by using the version v11b4 of the Heidelberg reference database, and no score above 0.3 towards a known entity was observed (no matching methylation classes with calibrated score≥0.3). Distribution of scores in the classification script showed a score of 0.2 towards the methylation class (anaplastic) pleomorphic xanthoastrocytoma (PXA). Additional methylation classes with even lower scores in decreasing order were GBM (MTCF_GBM), low-grade glioma and ganglioglioma (LGG_GG), control hemisphere (Control_HEMI), diffuse midline glioma H3 K27 M mutant (DMG_K27), and IDH glioma (MTCF_IDH_GLM).

Both variants were identified in the tumor. Notably, there was a skewed variant allele frequency of the *MEN1* frameshift variant (0.77) in both tumors sequenced. No additional somatic variants were detected in either *MEN1* or *CDKN1B*. To determine whether the second hit could be a large copy number variation (CNV) not detectable by WES (according to Knutson's two-hit theory), shallow whole-genome sequencing was performed on tumor DNA. The results pointed towards a deletion of one copy of chromosome 11, the chromosome harboring *MEN1*. Focused NGS identified a somatic BRAF p.Val600Glu (V600E) variant among the 20 genes investigated (Fig. 2a).

**Fig. 3:**
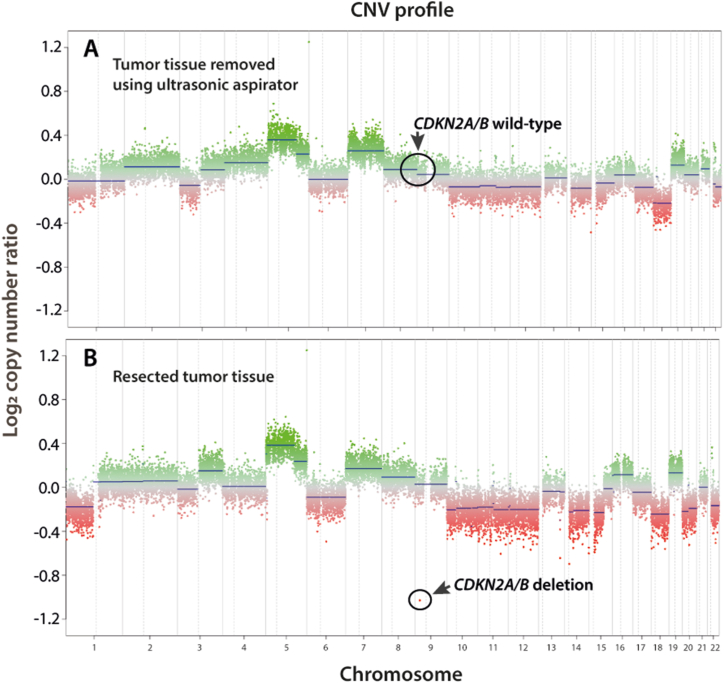
a No deletion of *CDKN2A/B* was found in the ultrasonic aspirate with similar, but less pronounced, trending of the CNV profile. **b** 850k-derived copy number variation (CNV) profile of the resected tumor showed marked chromosomal imbalances across the tumor genome and identified homozygous deletion of the *CDKN2A/B* locus.

Furthermore, the tumor did not achieve a match to a known entity in the reference set of the MolecularNeuropathology.org server database (v11b4) nor a score above 0.3. This new tumor entity could therefore not be classified based on its methylation signature [16]. The tumor entity with the highest score value of only 0.2 among the continuum of calibrated score values from DNA methylation profiling was PXA (Fig. 2b). CNV calculated from DNA methylation profiling data in overall concordance with CNV data from whole-genome sequencing showed intact 9p but focal loss of the *CDKN2A/B* genes, and only in tumor-enriched material (Fig. 3a and b). The methylation signature in this tumor case did not provide substantial information compatible with a known histologic diagnosis.

## 2 Discussion and conclusion

**Fig. 4:**
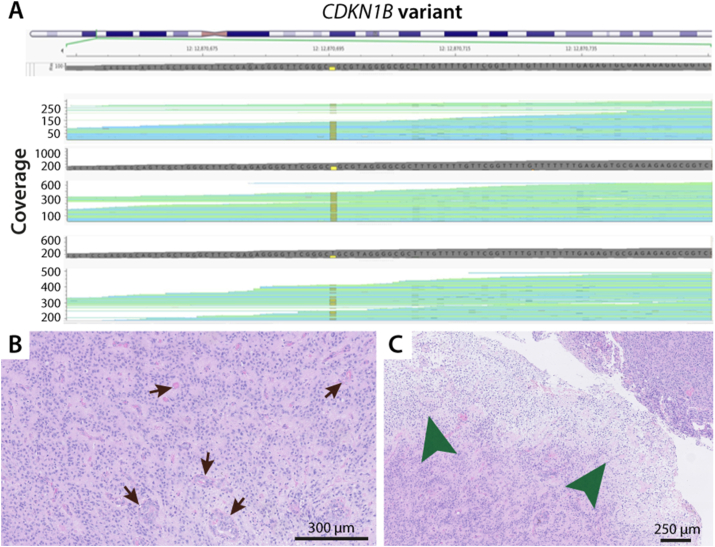
a *CDKN1B* variant identified by whole-exome sequencing with mean coverage 190 X. All coding regions were covered >30 X. **b** H&E-stained tumor biopsy (magnification × 10) showing high mitotic rate with intratumoral necrosis and true ependymal rosettes (black arrows). **c** At the same magnification and within the same specimen, the biphasic tumor is shown (indicated by green arrows) where the pleomorphic xanthoastrocytoma-like features can be detected.

Pellegata et al. identified a germline nonsense variant in the human *CDKN1B* gene, and their expanded pedigree analysis shows that the variant is associated with the development of a *MEN1*-like phenotype in multiple generations [21]. Germline *CDKN1B* variants are now known to cause the MEN 4 syndrome, and our group has recently reported that the variant is associated with hyperparathyroidism, pituitary tumors, neuroendocrine tumors, and unspecified tumors [7]. Brain tumor development in patients with MEN1 or MEN4 is a rare phenomenon, and the exact genetic cause for tumor development in these patients is not known [22]. In other words, multiple germline variants or polymorphism in MEN patients may potentially contribute to tumor development. Known germline or somatic *CDKN1B* variants have indeed not previously been associated with ependymoma- or PXA-like brain tumors. Furthermore, the *CDKN1B* variant (Fig. 4a) does not necessarily follow Knutson's two-hit theory with loss of heterozygosity [10], yet the variant can be related to more aggressive tumors [23,24]. This consideration is in line with our findings of tumor infiltration and mass effect on MRI (Fig. 1a–d), and high mitotic rate with intratumoral necrosis on histological analysis (Fig. 4 b-c). Shallow whole-genome sequencing on the tumor DNA showed deletion of one copy of chromosome 11 (chromosome harboring *MEN1*); this is a pathogenic variant with skewed variant allele frequency and deletion of one copy, i.e. the opposite of Knutson's two-hit theory.

**Fig. 5:**
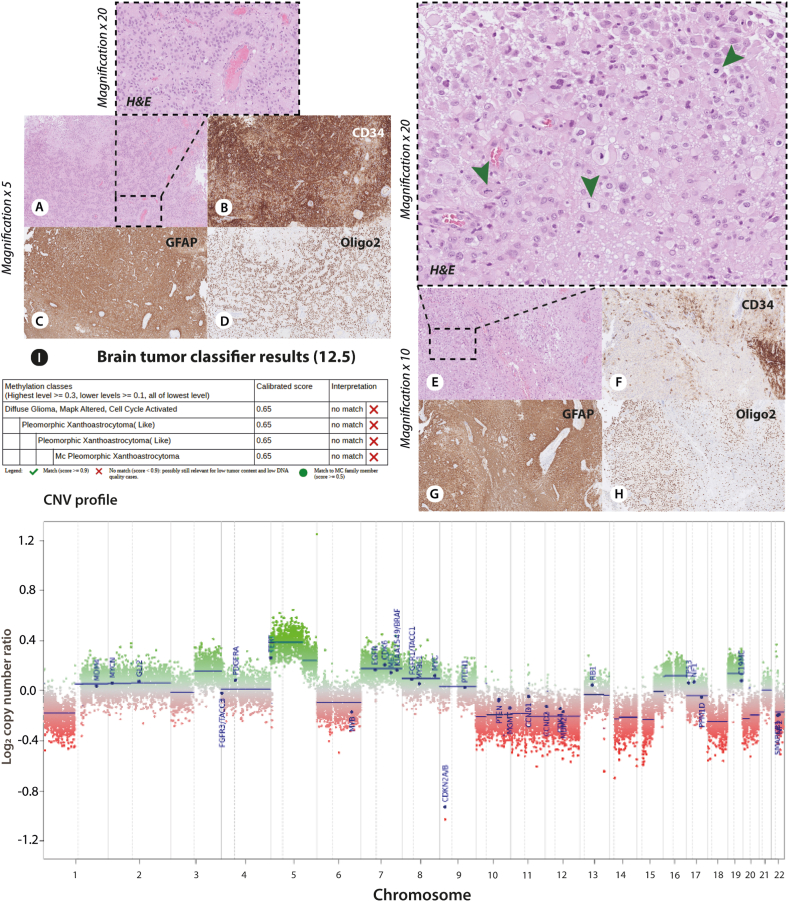
a H&E × 5 and at higher magnification (raised black box #1 × 20).; **b-d** Immunostaining showing strong CD34 signal, GFAP immunoreactivity and strong oligo2 expression supporting PXA diagnosis (magnification × 5). **e** Different areas shown reveal more syncytial growth to loosely cohesive aggregates with distinct cell membranes and abundant eosinophilic cytoplasm (magnification × 5). At higher magnification (raised black box #2 × 20) the pleomorphism is more pronounced with large vesicular nuclei, nucleoli, and dispersed mitoses. In some areas the cells detected are xanthomatous with vacuolation of the cytoplasm in addition of presenting more pleomorphic morphology with mitoses (green arrows). **f-h** Repeated immunohistochemistry gained from randomly chosen biopsies with the verification of variability in the positive CD34 expression, and with positive GFAP and oligo2 expression. **i** The PXA grade 3 diagnose is based on the histopathological and immunohistochemical features. By using the newest classifier (version 12.5) we found calibrated score of 0.65 (match score≥0.9), and the highest score was 0.84 (supplementary 1 and 2).

The tumor methylation classification for the presented case only favors PXA over other very different tumor entities, with normal brain hemisphere considered as baseline (classifier v11b4). Thus, a subtle hint towards PXA as differential diagnosis was derived from the methylation profiling. The PXA entity is rather diverse in its morphological appearance, and even cases without classical PXA appearance may be diagnosed as such with input from molecular data. The current difficult-to-classify case shows a complex CNV profile with numerous whole-chromosome gains and losses (Fig. 3). CNV features such as gain of chromosomes 5 and 7 and deletion of chromosomes 10, 14, and 22, in addition to deletion of *CDKN2A/B*, are shared with PXA, but the present CNV profile appears quite unique overall [16,25]. Our final histopathological diagnosis was made after immunohistochemistry (Fig. 5 a-h) and the most recent brain tumor classifier (v12.5) revealed v11b4 comparable methylation class and CNV profile (Fig. 5 i). The calibrated score for the ultrasonic aspirator tissue samples by using classifier v12.5 was 0.65, and for the resected tumor tissue samples 0.84 (Fig. 5 i and supplementary 1 and 2).

Identification of a putative modifying germline *CDKN1B* variant and a somatic deletion of *CDKN2/A* suggests an additional complex genetic contribution to brain neoplasia development in MEN1 syndrome. Moreover, this study confirms that brain tumors can potentially arise as part of MEN1 syndrome and should be recognized. Some of the clinical manifestations in the presented case are similar to those reported by Cuevas-Ocampo et al. [3]. However, we found no well-differentiated neuroendocrine tumors within the duodenal submucosa and/or pancreatic neuroendocrine tumors. In addition, we found histologic tumor features that distinguish from MEN1-associated ependymomas, which furthermore presents a methylation profiling not compatible with previously known brain tumors. We found that not only does this newly discovered tumor represent an PXA grade 3-like histotype and genotype, it also exhibits BRAF p.Val600Glu (V600E) variant as seen in PXA, and it has a highest score value of only 0.2 among a continuum of calibrated score values from DNA methylation profiling. Therefore, the methylation signature does not provide substantial information compatible with a known brain tumor diagnosis.

It brings limitations to the study that extension of the genetic analyses by including whole exome sequencing of all known coding genes as it could not be performed. This would include a risk of secondary findings and requires an extended signed informed consent from the patient according to Danish National Law. No additional mutated MEN associated genes screened with the above-mentioned gene panel were identified. The tumor did not achieve a calibrated score above 0.3 upon uploading to the MolecularNeuropathology.org. This was not followed by another approach such as t-Distributed Stochastic Neighbor Embedding (t-SNE) to determine the methylation-based class as MolecularNeuropathology.org was the only methylation classifier used. We did, however, extend our search for exact methylation match to a known brain tumor methylation profile, by using Brain Tumor Classifier 12.5. The data gained from this latest classifier version revealed no exact match to a known tumor entity (supplementary 1 and 2). From the CNV plot (Fig. 3b), chromosome 11 loss was not obvious, which contradicts with the WES and cannot be explained, and was not followed by fluorescence *in situ* hybridization analysis. The CNV plot suggested that this tumor may exhibit chromothripsis and intense genome rearrangements. Our WES analysis revealed such indication i.e., indeed compatible with chromothripsis. CDKN1B and MEN expression was not studied by immunohistochemistry, however, we demonstrate strong CD34, GFAP and Oligo2 expression with immunohistochemistry (Fig. 5 a-h). Hence, these data support the highest calibrated methylation score-finding of 0.84 (supplementary 2) and a final brain tumor diagnosis toward PXA. We convey that ependymoma or ependymoma-like tumors have already been shown to arise in the context of MEN1 syndrome. On the other hand, by this paper, we bring forward new data that raises the question whether PXA and PXA-like tumors can be associated with MEN1. We conjecture the importance of a second germline variant detected in CDKN1B. Expert sources (ClinVar) classify this variant as benign. The specific variant is indeed benign when classified form a monogenic perspective, however it has been described as a common modifier of MEN1 function. Therefore, the authors find it relevant to identify this potential modifying variant in this case with a very rare phenotypic presentation and not previously described brain tumor in MEN1.

In conclusion, we have followed the patient for four years since the primary post-operative control. He received proton therapy at the Danish Centre for Particle Therapy, five times per week for 2.5 months. Since then, we have not identified clinical deterioration, any recurrent DVT events, new neurological deficits or paraclinical signs of CNS malignant tumor relapses. The histopathological findings are compatible with PXA grade 3, and it should be noted that the identification of BRAF V600E mutation is common in other types of CNS tumors. Our findings could be co-incidental findings, however, in line with the report by Jannin et al. [26], the findings may also represent a novel rare type of cerebral tumor associated with MEN type 1. Currently, the surveillance program for MEN1 includes systematic screening for pituitary tumors and more evidence will be needed before extending with screening MEN1 for additional CNS tumors.

This study provides new insights into previously missing and unknown genetic MEN1 processes noted by Ozawa A. et al. [22]. Our findings are supported by Agarwal et al. [27] and suggest that the tumor genesis of MEN1-associated brain tumors (distinguished from ependymomas) does not necessarily follow Knutson's two-hit theory and can be triggered by *CDK* inhibitor germline variants. No clear molecular match to any known brain tumor entity was found in our case, but a *CDKN1B* pathogenic variant was detected along with deletion of *CDKN2/A,* hence these rare germline variants in cyclin-dependent kinase inhibitor genes may be considered as potential tumor markers for MEN1 patients with intracranial tumor manifestation. However, PXA grade 3 as differential pathology (PDP) and where we show low confidence score on methylation profiling combined with CDKN2A/2B deletion and BRAF V600E mutation, suggests PXA and not ependymoma.

## Funding

This research did not receive any specific grant from funding agencies in the public, commercial, or not-for-profit sectors.

## Patient consent

The patient provided written consent for the publication of this case report.

## Ethics statement

Heliyon & Cell Press Publishing ethics and publication guidelines were followed, in addition to the requirements of Danish National Committee on Health Research Ethics.

## Data availability

Data and materials supporting the results or analyses presented will be made available by the corresponding author upon reasonable request.

## Patient consent statement

The patient provided written consent for the publication of this case report.

## CRediT authorship contribution statement

**Halldór Bjarki Einarsson:** Writing – review & editing, Writing – original draft, Validation, Project administration, Methodology, Investigation, Formal analysis, Data curation, Conceptualization. **Anja Lisbeth Frederiksen:** Software, Methodology, Investigation, Formal analysis, Data curation, Conceptualization. **Inge Soekilde Pedersen:** Supervision, Software, Methodology, Investigation, Formal analysis, Data curation. **Marianne Schmidt Ettrup:** Validation, Methodology, Formal analysis. **Martin Wirenfeldt:** Validation, Methodology, Investigation, Data curation. **Henning Boldt:** Validation, Software, Resources, Methodology, Investigation, Formal analysis, Data curation. **Nina Nguyen:** Visualization, Formal analysis. **Marianne Skovsager Andersen:** Validation, Supervision. **Carsten Reidies Bjarkam:** Validation, Supervision. **Frantz Rom Poulsen:** Validation, Supervision, Project administration, Data curation, Conceptualization.

## Declaration of competing interest

The authors declare that they have no known competing financial interests or personal relationships that could have appeared to influence the work reported in this paper.

## Abbreviations

**ACMG**: The American College of Medical Genetics and Genomics
**BAM**: binary alignment map;
**BRAF**: B-Raf proto-oncogene, serine/threonine kinase
**BWA**: Burrows-Wheeler Aligner
**CD**: Cluster of Differentiation
**CDK**: cyclin-dependent kinase
**CDKN1B**: cyclin dependent kinase inhibitor 1B
**CDKN2/A**: cyclin dependent kinase inhibitor 2A
**CNV**: copy number variation
**CSF**: cerebrospinal fluid
**DVT**: deep venous thromboembolism
**EPIC**: emulsion, paired isolation, and concatenation
**FFPE**: formalin-fixed paraffin-embedded
**GBM**: glioblastoma multiforme
**GFAP**: Glial fibrillary acidic protein
**GG**: ganglioglioma
**gnomAd**: genome aggregation database
**HEMI**: hemisphere
**IDH**: isocitrate dehydrogenase
**INR**: international normalized ratio
**LGG**: low grade glioma
**MEN1**: multiple endocrine neoplasia type 1
**MEN4**: multiple endocrine neoplasia type 4
**MTCF**: methylation classes for
**NGS**: next-generation sequencing
**NOAC**: novel oral anticoagulant
**Olig2**: Oligodendrocyte transcription factor 2
**PDP**: Pleomorphic Xanthoastocytoma grade 3 differential pathology
**PHP**: primary hyperparathyroidism
**PXA**: Pleomorphic Xanthoastrocytoma
**VCF**: variant call format
**WES**: whole-exome sequencing
