# Supplementary material for: PDP type brain tumor in association with multiple endocrine neoplasia type 1
Source: Heliyon. 2024 Mar 12;10(6):e27418. doi: 10.1016/j.heliyon.2024.e27418 (PMC10951523; doi:10.1016/j.heliyon.2024.e27418)
Supplement: Multimedia component 1 [file mmc1.pdf]

Methylation profiling report

GERMAN CANCER RESEARCH CENTER IN THE HELMHOLTZ ASSOCIATION

Heidelberg University Hospital

MolecularNeuropathology.org

Supplier information

Sample identifier:	204294120129_R01C01
Sentrix ID:	204294120129_R01C01
Material type:	NA
Gender:	male
Supplier diagnosis:	ependymoma

Automatic prediction		
Array type:	EPIC	
Material type:	DNA-FFPE	✗
Gender:	male	✓

Legend: ✓ Ok | ! Supplier information or prediction not available | ✗ Warning, missmatch of prediction and supplier information

Brain tumor classifier results (12.5)

Methylation classes (Highest level >= 0.3, lower levels >= 0.1, all of lowest level)	Calibrated score	Interpretation	
Diffuse Glioma, Mapk Altered, Cell Cycle Activated	0.65	no match	✗
Pleomorphic Xanthoastrocytoma(Like)	0.65	no match	✗
Pleomorphic Xanthoastrocytoma(Like)	0.65	no match	✗
Mc Pleomorphic Xanthoastrocytoma	0.65	no match	✗

Legend: ✓ Match (score >= 0.9) ✗ No match (score < 0.9): possibly still relevant for low tumor content and low DNA quality cases. ● Match to MC family member (score >= 0.5)

Class descriptions

MC Pleomorphic xanthoastrocytoma: The "mc Pleomorphic xanthoastrocytoma" represents an astrocytic tumour with varied histology, primarily that of pleomorphic xanthoastrocytoma or anaplastic pleomorphic xanthoastrocytoma, but also including glioblastomas (particularly epithelioid glioblastoma) or with a ganglion cell-like differentiation appearing as 'anaplastic ganglioglioma'. There is no clear difference in methylation profiles between tumours histologically considered anaplastic or not. Location is typically supratentorial and often superficial (involving the leptomeninges). Most case arise in teenagers or young adults, with median age at diagnosis around 20-25 years. There is no apparent sex predilection. The majority of tumours in this mc harbor BRAF V600E mutations and homozygous deletions of CDKN2A/B. Cases lacking BRAF V600E typically show other alterations in the MAPK pathway, including NTRK family and RAF1 gene fusions. Some tumours of this mc also display TERT promoter mutations, which may be associated with a poorer prognosis.

Copy number variation profile

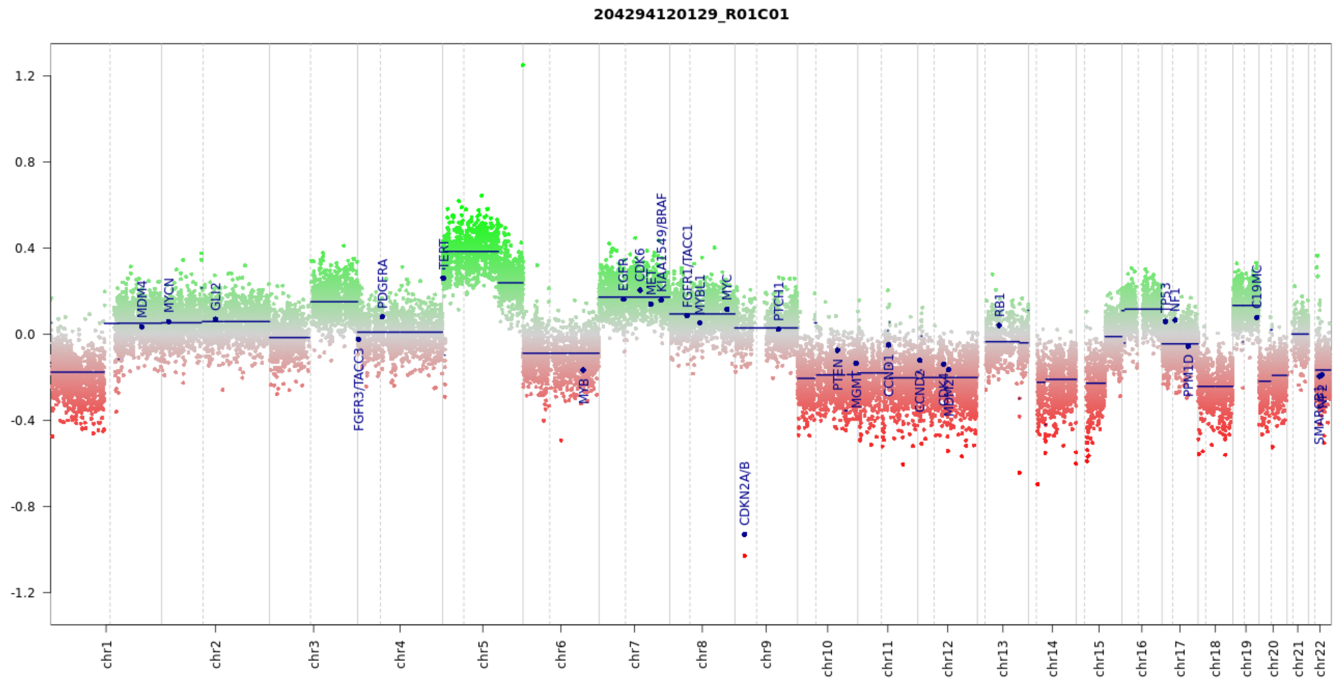

Depiction of chromosome 1 to 22 (and X/Y if automatic prediction was successful). Gains/amplifications represent positive, losses negative deviations from the baseline. 29 brain tumor relevant gene regions are highlighted for easier assessment. (see Hovestadt & Zapatka, http://www.bioconductor.org/packages/devel/bioc/html/conumee.html)

MGMT promotor methylation (MGMT-STP27)

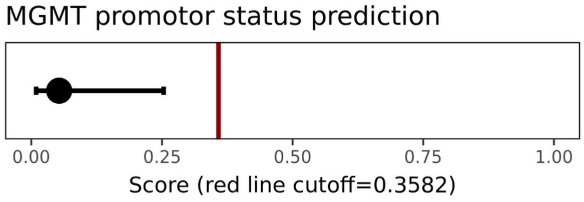

Status	Estimated	CI lower	CI upper
unmethylated	0.05329	0.00926	0.25319

(see Bady et al, J Mol Diagn 2016; 18(3):350-61)

Disclaimer

Classification using methylation profiling is a tool for research use only, it is not verified and has not been clinically validated and, therefore, must not be used for diagnostic purposes. This tool is not HIPAA compliant.

Run information

Report: report_website_mnp_brain_v12.5_sample (Version 1.0)

Task version:

Task	Version
idat_preprocess	2.0.1
idat_qc	2.0.1
idat_predictBrain	2.0.1
idat_rs_gender	2.0.1
idat_cnvp	3.0.1
idat_mgmt	2.0.1
report_website_mnp_brain_v11b4_research	2.1
report_website_mnp_brain_v11b4_sample	2.1
idat_predictBrain	12.5
report_website_mnp_brain_v12.5_sample	1.0
